# Beyond H37Rv: *Mycobacterium tuberculosis* pangenome structure and applications

**DOI:** 10.3389/fmicb.2025.1695567

**Published:** 2025-10-20

**Authors:** Andrea Monserrat Negrete-Paz, Gerardo Vázquez-Marrufo, Ma. Soledad Vázquez-Garcidueñas

**Affiliations:** ^1^División de Estudios de Posgrado, Facultad de Ciencias Médicas y Biológicas “Dr. Ignacio Chávez”, Universidad Michoacana de San Nicolás de Hidalgo, Morelia, Mexico; ^2^Centro Multidisciplinario de Estudios en Biotecnología, Facultad de Medicina Veterinaria, Universidad Michoacana de San Nicolás de Hidalgo, Morelia, Mexico

**Keywords:** pangenome, tuberculosis, core genome, pangenomic applications, MTBC

## Abstract

*Mycobacterium tuberculosis* (Mtb) is among the most successful bacterial pathogens, with multidrug-resistant strains posing significant challenges to global tuberculosis control. Traditional single-genome analyses, while essential for identifying strain-specific mutations, are limited in capturing the full spectrum of genetic diversity related to virulence, drug susceptibility, and transmission dynamics. Pangenomics examines the complete gene repertoire across all sequenced representatives of a species and addresses these limitations by enabling comprehensive, species-wide assessments of genetic variation. In this review, we summarize current knowledge of the Mtb pangenome, focusing on structural organization, methodological frameworks, and clinical applications. The Mtb pangenome exhibits a highly conserved genetic structure, with core genome estimates ranging from 1,166 to 3,767 genes, depending on the analytical thresholds and methodological approaches. Significant controversy regarding its classification as open or closed arises primarily from differences in computational pipelines (Roary, BPGA, Panaroo), core genome inclusion criteria (95%–100% presence), and dataset composition rather than fundamental biological disagreement. Despite these methodological challenges, pangenomic applications have demonstrated transformative potential in molecular epidemiology, drug resistance prediction, and virulence profiling. This perspective underscores a shift toward diversity-inclusive approaches, with integration of machine learning and standardization of analytical protocols identified as key priorities for future tuberculosis research and therapeutic innovation.

## 1 Introduction

Tuberculosis (TB), caused by the *Mycobacterium tuberculosis* complex (MTBC), remains one of the leading global infectious diseases. According to the WHO Global TB Report 2024, an estimated 10.8 million people developed TB in 2023. Although mortality rates have declined compared to the previous 2 years, TB has once again become the leading cause of death from a single infectious agent worldwide (World Health Organization [WHO], 2024). TB is primarily transmitted through airborne particles and is characterized as highly infectious and contagious, with a prolonged period of infectiousness (Martinez et al., 2017). Understanding the dynamics of transmission is crucial for outbreak control and for limiting the spread of the disease. While TB is distributed, it disproportionately affects low-income regions, particularly high-burden countries in Sub-Saharan Africa and South Asia (World Health Organization [WHO], 2024). Socioeconomic factors such as poverty, malnutrition, and poor living conditions are strongly associated with increased TB incidence (Lönnroth et al., 2009).

One of the most significant barriers to TB eradication and a major impediment to successful TB treatment is the alarming increase in multidrug-resistant (MDR) and extensively drug-resistant (XDR) strains (Dheda et al., 2024). Drug-resistant TB poses a critical threat to global public health, severely undermining treatment effectiveness and contributing to the persistence of the global epidemic (World Health Organization [WHO], 2024). Worryingly, resistance has emerged even against newly developed drugs (Zhou et al., 2025; Pym et al., 2016). This resistance is primarily driven by spontaneous mutations, followed by selective pressure that favors resistant strains (Gandhi et al., 2010). Beyond drug resistance, TB presents another persistent challenge: its remarkable ability to remain latent in human hosts. It is estimated that approximately one quarter to one-third of the global population (around two billion people) harbor latent TB. Among these, only 5%–10% will develop active diseases during their lifetime (World Health Organization [WHO], 2024). The ability of *Mycobacterium tuberculosis* (Mtb) to persist in a dormant state, combined with its propensity for developing drug tolerance and resistance, contributes to TB relapses, a growing concern for global elimination programs (Bhalla and Nanda, 2024).

Over the past three decades, molecular epidemiology has significantly enhanced our understanding of TB transmission dynamics and evolutionary biology, thereby informing public health strategies (Guerra-Assunção et al., 2015). Whole-Genome Sequencing (WGS) has revolutionized TB research, offering unmatched genetic resolution compared to traditional genotyping methods such as IS6110-RFLP, spoligotyping, and MIRU-VNTR (Ng et al., 2024). These earlier methods, though valuable, suffered from limitations including low discriminatory power, labor-intensive protocols, and poor reproducibility when applied to closely related strains (Guerra-Assunção et al., 2015; Couvin et al., 2025).

## 2 From single genome analysis to pangenomics

The initial application of WGS in TB research focused primarily on comparative analyses between individual clinical strains and the H37Rv reference genome. This approach yielded insights into strain-specific mutations, particularly those associated with drug resistance and virulence (Cole et al., 1998; Zheng et al., 2008). However, despite its revolutionary impact, this genome-centric methodology, presented intrinsic limitations in capturing the broader genetic landscape and evolutionary dynamics of Mtb population at regional and global scales. While single genome analyses were instrumental in identifying key genetic determinants of pathogenicity and resistance, they offered narrow primarily strain-specific perspectives that reflected only a limited portion of the species’ overall genomic diversity. Each sequenced strain constituted a temporal snapshot of the Mtb genome, and although successive studies incrementally expanded our understanding of its genetic repertoire, the limitations of single-genome analyses became evident (Vernikos et al., 2015). Furthermore, comparative studies restricted to pairwise alignments frequently failed to capture larger-scale patterns of gene presence, absence, and functional distribution that define the species (Vernikos et al., 2015; Dunn et al., 2018).

The recognition of the limitations of traditional genomic approaches has catalyzed a paradigm shift toward pangenomics. This analytical framework encompasses the entire gene repertoire of a bacterial species across all its sequenced representatives (Tettelin et al., 2005). By integrating data from multiple genomes simultaneously, pangenomics offers a comprehensive overview of species-wide genetic content, revealing patterns of gene conservation, acquisition, and loss that remain undetectable when analyzing genomes in isolation, as a pairwise comparison with a reference genome.

In the case of Mtb, the transition to pangenomic research has proven particularly valuable due to the pathogen’s clinical relevance and the increasing availability of high-quality genome sequences from diverse geographical regions and evolutionary lineages. This framework enables researchers to systematically examine how genetic diversity correlates with phenotypic traits, such as virulence, drug susceptibility, and host adaptation across the MTBC. Furthermore, it provides critical insights into the evolutionary forces shaping the genetic architecture of the species, advancing both our fundamental understanding of TB biology and its practical applications in diagnostics, treatment, and epidemiological monitoring.

As our understanding of how genomic diversity affects mycobacterial virulence and transmissibility continues to deepen (Coscolla and Gagneux, 2014), this comprehensive genetic perspective becomes increasingly vital. Thus, the pangenomic approach represents not only a methodological advance but also a fundamental reconceptualization of how Mtb is studied as a genetically diverse pathogen with profound implications for global public health.

## 3 The *Mycobacterium tuberculosis* pangenome: definition and conceptual framework

### 3.1 Pangenome architecture in *M. tuberculosis*

The Mtb genome is known for its high degree of conservation, exhibiting limited genetic variability across strains (Sreevatsan et al., 1997). This genomic stability is largely attributed to the organism’s clonal nature and the near absence of horizontal gene transfer, which restricts opportunities for large scale genomic diversification (Becq et al., 2007).

The pangenome of MTBC is organized into distinct functional compartments based on gene frequency distribution across strains. The core genome, comprising genes present in all analyzed strains, encodes essential functions such as basic metabolism, cell wall biosynthesis, and key cellular processes (Tettelin et al., 2005). Recent estimates place the size of the Mtb core genome between 3,032 and 3,767 genes, reflecting a relatively conserved genetic backbone required for viability and virulence (Espinoza et al., 2025; Morey-León et al., 2025). The soft-core genome includes genes found in most strains (≥95%), typically encompassing near universal functions within the species (Periwal et al., 2015). In contrast, the accessory genome comprises genes present in a subset of strains, often reflecting lineage-specific, geographically restricted, or phenotype-associated traits, such as drug resistance or virulence factors (Dar et al., 2020). Large-scale analyses of clinical isolates underscore the core genome’s role as a blueprint of essential pathogenic functions, offering key insights into the genetic architecture necessary for infection establishment and maintenance (Periwal et al., 2015). The highly conserved foundation contrasts markedly with the accessory genome’s variability, which contributes to lineage-specific adaptations and regional epidemiological dynamics (Chekesa et al., 2024). Such genomic duality highlights an evolutionary process wherein the accessory genome serves as a reservoir of genetic innovation, promoting adaptability to diverse host environments and selective pressures, including the emergence of antimicrobial resistance and enhanced virulence (Espinoza et al., 2025).

### 3.2 Comparative pangenomic context: *M. tuberculosis* among intracellular pathogens

To contextualize the distinctive features of the Mtb pangenome, it is instructive to compare its genetic architecture with that of other intracellular bacterial pathogens. Such comparative analyses aid in revealing both convergent evolutionary patterns and species-specific adaptations that delineate Mtb’s unique position within the broader spectrum of intracellular pathogenesis. Pangenome size and conservation levels vary widely among intracellular pathogens. For instance, the facultative intracellular bacterium *Salmonella enterica* possesses a core genome of approximately 2,800 genes within a compact pangenome of ∼10,000 gene families, indicating genomic plasticity in contrast to its close relative *E. coli*, which exhibits continuous expansion of its genetic repertoire (Jacobsen et al., 2011). In contrast, *Legionella pneumophila*, demonstrates a core genome of 1,979 genes, but markedly greater genetic diversity reflecting its adaptation to diverse environmental niches and extensive horizontal gene transfer (D’Auria et al., 2010). Similarly, *Listeria monocytogenes*, another facultative intracellular pathogen, shows moderate genetic diversity with lineage-specific adaptations across its three major phylogenetic groups (den Bakker et al., 2010). Within this comparative framework, Mtb stands out by maintaining a larger and conserved core genome comprising over 3,000 genes (Espinoza et al., 2025; Morey-León et al., 2025; Behruznia et al., 2025; Bundhoo et al., 2024) than other intracellular pathogens such as *Salmonella enterica* (∼2,800 genes), *Legionella pneumophila* (1,979 genes), or *Listeria* spp. (2,032 core genes) (Jacobsen et al., 2011; D’Auria et al., 2010; den Bakker et al., 2010). This suggests that Mtb obligate pathogenic lifestyle demands retention of a broader set of essential functions across all strains, allowing genomic variability and reduced tolerance for gene loss compared to facultative pathogens, which rely on greater inter-strain plasticity to adapt to variable environmental conditions.

These evolutionary constraints and their functional consequences become clearly evident through pangenomic comparisons. Evidence indicates that patterns of genetic diversity are closely linked to a pathogen’s ecological niche and evolutionary history, with gene frequency function relationships showing across species conservation (Hyun et al., 2022). In this context, the restricted genetic diversity of Mtb reflects its specialization for human-to-human transmission and limited environmental survivability, unlike *L. pneumophila*, which relies on broad genetic diversity for environmental adaptation, or *S. enterica*, which navigates a dual lifestyle between host and the environment. Thus, the Mtb pangenome architecture emerges as a distinct evolutionary strategy: a highly conserved genome with minimal accessory content, optimized for stable pathogenicity within a specific host range. This architecture contrasts with the dynamic and open pangenomes characteristic of a facultative intracellular bacterium, underscoring the unique evolutionary trajectory of Mtb. Nonetheless, the full extent and implications of this genomic structure remain to be researched.

## 4 The open vs. closed pangenome controversy in *M. tuberculosis*

A critical question in understanding Mtb genomic architecture is whether its pangenome follows an open or closed model, an issue with significant implications for microbial evolution, epidemiology, and the emergence of drug resistance.

The classification of the Mtb pangenome as open or closed has been a subject of ongoing debate, representing one of the most contentious aspects of TB genomics. This controversy arises from differences in analytical approaches, dataset composition, and the interpretation of mathematical models used to predict pangenome dynamics (Tettelin et al., 2008; Rouli et al., 2015; Marin et al., 2025). Pangenome openness is typically assessed through pangenome saturation curves, which plot the cumulative number of unique genes identified (*y*-axis) against the number of genomes analyzed (*x*-axis) (Tettelin et al., 2005).

### 4.1 Evidence of an open pangenome

An open pangenome is characterized by a substantial accessory gene pool and high inter-strain genomic diversity. In Mtb, this diversity arises predominantly through structural genomic alterations such as deletions, duplications, and rearrangements, rather than horizontal gene transfer (Periwal et al., 2015; Marin et al., 2025; Zakham et al., 2021). Mathematically, an open pangenome is inferred when gene accumulation curves continue to rise without plateauing, indicating ongoing gene discovery with the inclusion of additional genomes (Tettelin et al., 2005, 2008). A Heaps’ alpha value (α) < 1 further supports this model suggesting a non-saturating, theoretically unbounded pangenome (Espinoza et al., 2025). In contrast, a closed pangenome is defined by gene loss and deletion events rather than acquisition, with saturation curves reaching a plateau, indicating that most genes have already been identified (Behruznia et al., 2025).

Compelling evidence supports the open pangenome architecture in Mtb. Mathematical models based on saturation curves, derived from datasets encompassing 96 to 500 genomes, consistently reveal non-saturating trajectories, implying ongoing gene discovery (Periwal et al., 2015; Negrete-Paz et al., 2023). Empirical data corroborate these findings with geographically diverse strain collections exhibiting substantial accessory gene variation (Morey-León et al., 2025; Chekesa et al., 2024; Yang et al., 2018). Long-read sequencing technology has further revealed pangenomes containing up to 4,325 total genes, of which 558 are accessory, highlighting a degree of genomic variability incompatible with a closed model (Espinoza et al., 2025). This diversity is driven by multiple mechanisms, including copy number variation, structural genome modeling, and lineage-specific patterns of gene presence or absence, which collectively contribute to pangenome expansion (Bhalla and Nanda, 2024; Yang et al., 2018). The convergence of these mathematical inferences, empirical observations, and mechanistic insights from highly diverse strain datasets supports the classification of Mtb as possessing an open pangenome architecture.

### 4.2 Evidence for a closed pangenome

Despite growing support for an open pangenome in Mtb, a substantial body of literature suggests the opposite, a closed or nearly closed pangenomic structure. Such findings emerge from independent research groups employing varied analytical approaches and strain datasets yet consistently reporting limited genetic diversity and extensive genomic conservation (Behruznia et al., 2025; Zakham et al., 2021; Silva-Pereira et al., 2024). Biological characteristics intrinsic to Mtb support the closed pangenome model. Unlike many bacteria with open pangenomes maintained by active horizontal gene transfer, Mtb displays minimal to no evidence of such events, lacks plasmids, and exhibits a strong clonal population structure (Derbyshire and Gray, 2014). Consequently, gene acquisition plays a negligible role in shaping its genomic diversity; instead, gene loss is the predominant mechanism of genomic variation within the MTBC (Silva-Pereira et al., 2024; Costa et al., 2020). Mathematical analyses further reinforce this view. Pangenome curves generated from various datasets exhibit plateau formation, and power-law regression models yield coefficients supporting minimal potential for future expansion (Dar et al., 2020). Empirically, constrained pangenomic profiles have been reported in comprehensive analyses of 324 complete genomes spanning all major lineages with only modest accessory gene content identified (Behruznia et al., 2025). Similarly, studies of 420 epidemiologically diverse strains identified only 85 novel genes beyond the reference genome, underscoring a limited capacity for genome expansion (Zhou et al., 2025). Additional support comes from showing that core genome sizes are relatively small (e.g., 1,166 conserved genes in human-adapted MTBC strains), and that observed diversification is largely driven by phylogenetic inheritance rather than acquisition processes (Zakham et al., 2021; Silva-Pereira et al., 2024). Machine learning approaches have further confirmed these patterns by detecting genomic signatures consistent with constrained rather than expansive, evolutionary dynamics (Kavvas et al., 2018). Moreover, some apparent signs of pangenome expansion in prior studies have been attributed to artifacts such as poor genome assembly quality or inconsistent gene annotation criteria (Marin et al., 2025), casting doubt on the robustness of some claims of continuous gene discovery.

The classification of the Mtb pangenome as open or closed has profound biological and clinical implications. An open pangenome would imply that the species retains the capacity for continuous diversification. This scenario suggests greater adaptive potential, particularly in response to antibiotic pressure, as accessory genes or structural rearrangements could generate new resistance determinants or enhance tolerance mechanisms (Periwal et al., 2015; Yang et al., 2018; Espinoza et al., 2025). Likewise, an open model would support the notion that virulence traits may continue to diversify across lineages, contributing to heterogeneous clinical phenotypes and potentially complicating vaccine or drug development strategies. From an evolutionary perspective, an open pangenome aligns with long-term adaptability, allowing Mtb to persist under fluctuating host and environmental pressures. In contrast, a closed pangenome underscores the remarkable evolutionary stability of the MTBC, where genomic innovation is limited and adaptation arises primarily through point mutations and gene loss rather than acquisition of novel functions (Silva-Pereira et al., 2024). This model explains why drug resistance in this pathogen is almost exclusively mutation-driven, often involving well-characterized chromosomal targets. A closed pangenome also suggests that virulence factors are largely fixed, which may explain the conserved pathogenesis mechanisms across global lineages despite geographical and host diversity (Zakham et al., 2021; Dar et al., 2020). Ultimately, whether the Mtb pangenome is truly open or closed shapes our expectations for its long-term evolutionary trajectory, the mechanisms by which resistance and virulence emerge, and the strategies required for global TB control. Bridging methodological variation with biological interpretation is therefore critical to fully leverage pangenomics for both basic and translational research.

### 4.3 Methodological factors contributing to divergent pangenome classifications

The ongoing debate over whether Mtb pangenome is open or closed is significantly influenced by methodological heterogeneity across studies. Divergent classifications are often not the result of biological inconsistency but rather stem from differences in analytical tools, genome quality, sequencing platform, threshold criteria, and data set composition. Understanding these technical determinants is essential for reconciling interpretations and establishing a standardized framework for pangenomic analysis.

#### 4.3.1 Analytical tools and processing parameters

A principal source of variation in pangenome architecture arises from the choice of computational tool used for pangenome construction. Software such as Roary (Page et al., 2015), BPGA (Chaudhari et al., 2016), and Panaroo (Tonkin-Hill et al., 2020) apply distinct algorithms for ortholog detection, gene clustering, and similarity thresholding. Such differences lead to substantially divergent estimates of pangenome size and composition even when applied to identical datasets. In Mtb, these tool-dependent biases manifest as distinct patterns: Roary’s conservative similarity thresholds tend to fragment repetitive gene families, artificially inflating accessory genome estimates, while BPGA’s clustering algorithms may inappropriately merge divergent PE/PPE family members, and Panaroo’s stringent error correction, though reducing false positives, can occasionally exclude genuine rare variants characteristic of highly clonal populations (Marin et al., 2025; Behruznia et al., 2025). The heterogeneity observed in reported pangenome sizes (Table 1) illustrates the methodological dependence of *M. tuberculosis* pangenome estimates. To contextualize these differences, Table 2 provides a comparative overview of the main pipelines, highlighting their strengths, limitations, and applicability to clonal pathogens.

**TABLE 1:** Summary of pangenome studies conducted on *Mycobacterium tuberculosis* complex (MTBC).

Dataset size	Core	Accessory	Open/closed	Tools	Application	Reference
96 MTBC	2,066	6,033	Open	Prodigal, CD-HIT, BLAST	Identification of essential core genes	Periwal et al., 2015
146 *M. tuberculosis*	Not specified	Not specified	Not specified	PANPASCO	Molecular epidemiology	Jandrasits et al., 2019
88 *M. tuberculosis*	3,032	3,671	Near to close	Roary	Molecular epidemiology	Morey-León et al., 2025
	3,104	1,426		BPGA		
233 MTBC	3,116	947	Closed	PGAP	Molecular epidemiology	Silva-Pereira et al., 2024
442 *M. tuberculosis*	2,754	Not specified	Near to close	BPGA	Therapeutic target discovery and drug resistance	Khan et al., 2024
88 *M. tuberculosis*	3,104	936	Closed	Panaroo	Molecular epidemiology	Morey-León et al., 2025
110 *M. tuberculosis*	3,767	558	Open	Panaroo	Therapeutic target discovery and virulence	Espinoza et al., 2025
264 *M. tuberculosis*	3,241	2,109	Not specified	Not specified	Virulence and pathogenicity	Bundhoo et al., 2024
1,595 *M. tuberculosis*	2,803	Not specified	Closed	Machine learning	Therapeutic target discovery and virulence	Kavvas et al., 2018
183 MTBC	1,166	5,870	Near to close	BLASTP	Molecular epidemiology	Zakham et al., 2021
121 *M. tuberculosis*	3,698	4,237	Open	Spine, AGEnt, ClustAGE	Virulence and pathogenicity	Rufai et al., 2020
47 *M. tuberculosis*	3,566	1,196	Open	Prokka, Prodigal, BLASTP, GET_HOMOLOGUES, BPGA	Molecular epidemiology	Hurtado-Páez et al., 2023
33 *M. tuberculosis*	3,679	2,086	Open	PGAP, PanGP	Virulence and pathogenicity	Yang et al., 2018
150 *M. tuberculosis*	1,251	Not specified	Not specified	BPGA	Therapeutic target discovery and drug resistance	Dar et al., 2020
75 *M. tuberculosis*	3,270	1,667	Open	BLASTP, GET_HOMOLOGUES, BPGA	Molecular epidemiology	Chekesa et al., 2024
490 *M. tuberculosis*	2,231	3,729	Near to close	Roary, Panaroo	Virulence and pathogenicity	Negrete-Paz et al., 2023
2,184 *M. tuberculosis*	3,784	1,109	Open	Panaroo	Therapeutic target discovery and drug resistance	Bhalla and Nanda, 2024
151 *M. tuberculosis*	3,833	3,879	Not specified	Panaroo, PpanGGolin, Roary, Pangene	Methodological review	Marin et al., 2025
335 MTBC	3,639	1,008	Closed	Panaroo, Pangraph	Molecular epidemiology	Behruznia et al., 2025
420 *M. tuberculosis*	3,438	Not specified	Closed	Prokka, GET_HOMOLOGUES	Molecular epidemiology	Zhou et al., 2025

MTBC, *Mycobacterium tuberculosis* complex.

**TABLE 2:** Comparative overview of major pangenome construction pipelines and their suitability for *Mycobacterium tuberculosis*.

Tool	Approach/algorithm	Strengths	Limitations	Suitability for *M. tuberculosis*
Roary (Page et al., 2015)	CD-HIT BLASTP clustering of orthologs	Fast, scalable (>1,000 genomes) Widely adopted Strong community support	Overestimates accessory genome by fragmenting paralogs	Commonly used, but risk of inflating diversity
BPGA (Chaudhari et al., 2016)	BLAST/USEARCH-based ortholog clustering	Integrates functional annotation Flexible analyses	May merge divergent paralogs Less optimized for clonal pathogens	Useful for functional profiling Moderate accuracy in paralog-rich genomes
Panaroo (Tonkin-Hill et al., 2020)	Graph-based gene clustering Error correction	Reduces false positives Robust against assembly/annotation errors Ideal for clonal species	Can exclude true rare variants Higher computational cost	Highly suitable Minimizes artificial diversity in clonal MTBC
PGAP (Zhao et al., 2012)	OrthoMCL-based ortholog clustering / gene family assignment	Sensitive orthology assignment Strong statistical rigor	Computationally demanding Less scalable	Useful for medium-sized datasets and reference-based comparisons
PanX (Ding et al., 2018)	Phylogenetic-aware graph clustering	Integrates evolutionary context Interactive visualization of gene gain/loss	Less efficient for very large datasets Limited adoption	Suitable for evolutionary/phylogenetic interpretation of MTBC

Another critical factor involves the criteria used to define the core genome. Thresholds for gene presence across strains vary considerably: some studies adopt strict 100% inclusion criteria (Zhou et al., 2025; Yang et al., 2018), while others use relaxed thresholds ranging from 95% to 99% presence across analyzed strains (Morey-León et al., 2025; Periwal et al., 2015; Behruznia et al., 2025). These disparities in threshold decisions have a profound impact on the classification of genes as core or accessory. For instance, Periwal et al. (2015) found that a 95% presence threshold maximized core genome representation of essential functions, whereas Morey-León et al. (2025) employed a 99% threshold to refine core gene inclusion. Adjusting the threshold from 95% to 100% can reclassify hundreds of genes, dramatically altering pangenome size and the inferred open or closed status (Periwal et al., 2015; Morey-León et al., 2025).

These wide variation in Mtb core genome estimates across studies ranges from as few as 1,166 conserved genes in strictly human-adapted species (Zakham et al., 2021; Silva-Pereira et al., 2024) to over 3,767 genes in broader, more inclusive analyses (Espinoza et al., 2025; Morey-León et al., 2025). Such variability underscores the sensitivity of pangenome architecture to analytical parameters.

#### 4.3.2 Genome quality and assembly standards

Genome assembly quality is a fundamental determinant of pangenomic accuracy, with sequencing platform choice exerting a profound influence on pangenome estimates. Draft assemblies are prone to fragmentation, misassembly, and annotation artifacts, which can artificially inflate estimates of accessory genomes and obscure the accurate gene content (Zhou et al., 2025). Platform-specific characteristics compound these challenges through distinct error profiles and assembly biases that differentially impact pangenome inference. Short-read sequencing technologies, while offering high accuracy and throughput, systematically fragment repetitive genomic regions essential for accurate pangenome reconstruction. Illumina-based assemblies frequently break at PE/PPE gene clusters and IS6110 insertion sites, creating artificial gene truncations (Marin et al., 2025). These fragmentation artifacts are particularly problematic for Mtb pangenome studies, where repetitive sequences comprise a significant genomic content, yet are critical for accurate strain differentiation. On the other side, long-read sequencing platforms address many assembly limitations but introduce distinct biases affecting pangenome estimates. Oxford Nanopore technologies demonstrate superior performance for repetitive sequence resolution but exhibit higher indel error rates that can create false gene variants during annotation (Behruznia et al., 2025). PacBio SMRT sequencing offers improved accuracy for complex genomic architectures yet requires higher coverage depths to achieve comparable gene detection sensitivity. Coverage depth effects are particularly pronounced in pangenome studies, where insufficient sequencing depth can systematically underrepresent low-abundance genes or create false absence calls that skew core-accessory genome classifications.

Platform choice also influences downstream analytical pipelines through assembly contiguity effects. Highly fragmented short-read assemblies may require different clustering parameters compared to complete long-read assemblies, complicating comparative analyses across mixed-platform datasets (Marin et al., 2025). Analysis limited to complete high-quality genomes may underestimate pangenome diversity by excluding rare or lineage-specific genes lost during assembly curation. In contrast, inclusion of draft assemblies introduces systematic inflation through technical artifacts.

#### 4.3.3 Dataset composition and representativeness

Beyond computational and technical factors, the composition and diversity of analyzed datasets also impact pangenome interpretation. Geographic sampling bias can skew accessory genome estimates by overrepresenting strain specific elements that do not reflect global patterns (Morey-León et al., 2025; Chekesa et al., 2024). Likewise, dataset size and phylogenetic breadth are crucial. Smaller or phylogenetically homogeneous datasets often support closed pangenome models due to limited genetic diversity, while larger, more diverse collections tend to reveal great accessory gene content and support an open architecture (Dar et al., 2020; Silva-Pereira et al., 2024).

### 4.4 Biological factors affecting pangenome inference in *M. tuberculosis*

**FIGURE 1:**
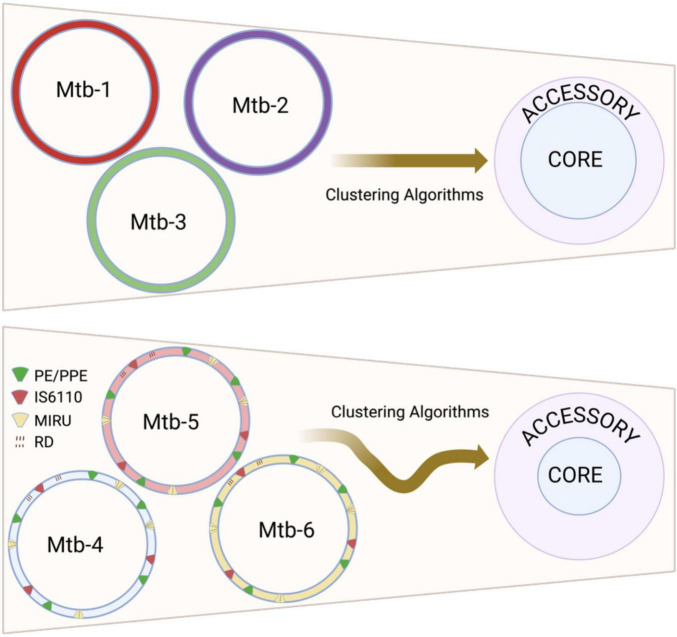
Impact of repetitive sequences on reconstructing the *Mycobacterium tuberculosis* pangenome. The repetitive sequence features of *M. tuberculosis* include direct repeats (DR), Mycobacterial Interspersed Repetitive Units (MIRU), the insertion sequence 6110 (IS6110), and the PE/PPE gene family. Clustering algorithms struggle to accurately identify homologous sequences in these repetitive regions during pangenome reconstruction. These inaccuracies tend to falsely inflate the accessory genome (lower panel) compared to genomes without such repetitive sequences (upper panel). Each circle labeled “Mtb” represents genomes from different *M. tuberculosis* strains. Created in BioRender. Vázquez-Marrufo (2025) https://BioRender.com/kzarxxt.

The intrinsic biological characteristics of Mtb create unique challenges for accurate pangenome inference that extend beyond purely methodological considerations. The species’ clonal population structure and restricted genetic diversification mechanisms render pangenome estimates particularly vulnerable to technical artifacts, as genuine biological variation cannot be easily distinguished from methodological noise (Bolotin and Hershberg, 2015). Additionally, besides de bacterial variable number tandem repeat units (VNTR), the prevalence of specific repetitive sequences in the Mtb genome, including the direct repeats (DRs), the mycobacterial intersperse repetitive units (MIRUs), the PE/PPE gene families and IS6110 elements (Arnold, 2007; Delogu et al., 2017), systematically complicates clustering algorithms (Figure 1), while the predominance of gene loss over acquisition in Mtb evolution further constrains the biological context available for validating apparent genetic diversity (Yang et al., 2018; Silva-Pereira et al., 2024). This biological constraint amplifies the impact of technical decisions on estimates of pangenome architecture. In species with active gene acquisition, spurious gene detection can often be identified through phylogenetic incongruence or atypical sequence characteristics. However, Mtb’s evolutionary history, which is primarily characterized by chromosomal rearrangements and deletions, provides no such comparative framework, making every apparent genetic variant potentially legitimate from a biological perspective. Consequently, methodological choices regarding clustering parameters, similarity thresholds, and quality control measures exert disproportionate influence on final pangenome estimates, as biological plausibility alone cannot serve as a filter for technical artifacts.

In summary, the classification of the Mtb pangenome is highly sensitive to both biological constraints and methodological heterogeneity. The species’ intrinsic characteristics, including clonal structure, repetitive sequences, and reductive evolution, create a genomic context where technical artifacts are difficult to distinguish from genuine variation. Compounding these biological factors, differences in computational tools, parameter thresholds, genome quality, and dataset composition can each independently and often synergistically affect the interpretation of pangenome structure. These combined sources of biological and methodological variability underscore the urgent need for standardized protocols that account for species-specific characteristics to ensure reproducibility and comparability across studies. The following section outlines systematic approaches for robust pangenome construction that address these challenges.

## 5 Pangenome construction: from raw data to biological insights

Pangenome construction in bacterial genomics has evolved along two principal methodological paradigms: clustering-based and graph-based approaches. Clustering -based methods identify homologous gene families across genome assemblies and classify them into presence-absence matrices, facilitating large scale comparative analyses with computational efficiency and categorical distinction of core and accessory genes. However, these approaches traditionally focus exclusively on protein-coding sequences, potentially overlooking regulatory elements, non-coding RNAs, and intergenic regions that contribute to phenotypic diversity and evolutionary adaptation (Vernikos et al., 2015). In contrast, graph-based methods model the pangenome as an interconnected network, preserving structural variation, allelic diversity, and synteny across both coding and non-coding genomic content. This paradigm offers high resolution for detecting complex evolutionary events such as inversions, duplications, and recombination features often missed by binary matrix approaches (Tonkin-Hill et al., 2020; Garrison et al., 2018; Hickey et al., 2020). The inclusion of non-coding content through whole-genome approaches reveals substantially higher genomic diversity, with recent studies reporting approximately 22% variable genomic content when intergenic regions and partial gene deletions are included, compared to approximately 10% accessory content identified through protein coding analysis (Behruznia et al., 2025). The choice between analyzing exclusively coding sequences versus including non-coding genomic content has implications for Mtb pangenome construction, where genetic diversity is primarily driven by large sequence polymorphisms and regions of difference that often encompass both coding and regulatory sequences, as previously stated. Protein coding-based analyses may fragment these evolutionary units, potentially underestimate the functional impact of structural variants or missing regulatory mutations that influence gene expression without altering protein sequences. This methodological limitation can lead to systematic underrepresentation of lineage-specific adaptations, as regulatory variations in promoter regions of core genes remain undetected despite their potential phenotypic consequences (Behruznia et al., 2025). Conversely, including non-coding content provides a more comprehensive view of genomic diversity but introduces analytical challenges in establishing appropriate similarity thresholds for intergenic regions and distinguishing genuine regulatory variation from sequencing artifacts. The selection between paradigms depends on research objectives and analytical priorities: clustering-based methods offer efficiency for large-scale population genetics, functional gene surveys, and studies where structural variants manifest as clear gene presence/absence patterns, while graph-based approaches provide enhanced resolution for detailed structural characterization, particularly when analyzing complex rearrangements, partial deletions, or regulatory modifications that span multiple genomic elements (Garrison et al., 2018; Tonkin-Hill et al., 2020). Despite the enhanced resolution of graph-based models for certain structural analyses, clustering-based strategies remain the standard in bacterial pangenomics due to their scalability, established analytical pipelines, and compatibility with statistical frameworks (Tettelin et al., 2005; Rouli et al., 2015). Given their prevalence, the following sections outline the standardized workflow, species-specific considerations, and gene prediction methodologies that underpin clustering-based Mtb pangenome construction.

### 5.1 General workflow overview

Clustering-based pangenome construction follows a standardized computational pipeline comprising: (i) genome collection and quality assessment, (ii) gene prediction and annotation, (iii) homology detection and clustering, (iv) presence-absence matrix generation, and (v) statistical and functional characterization (Medini et al., 2005). Homologous gene families are identified across genome assemblies and classified into core, accessory, and unique gene sets according to frequency distribution thresholds (Tettelin et al., 2005). This framework enables quantification of genomic diversity and functional partitioning at the species level.

### 5.2 Dataset requirements and species-specific considerations

Robust pangenome construction of Mtb requires stringent dataset quality and thoughtful phylogenetic representation due to its highly clonal nature and restricted gene flow. Assemblies should fall within the expected genome size range (4.2–4.5 Mb) with N50 values > 100 kb, and contamination levels < 1% as assessed by tools such as CheckM. Complete genome assemblies are strongly preferred as draft assemblies compromise orthology detection (Zhou et al., 2025). If draft genomes are used, stringent filters must be applied, including a minimum contig length of ≥1 kb, a maximum contig number of <200, and a completeness of ≥95% to maintain analytical integrity (Parks et al., 2015).

Moreover, the pronounced phylogeographic structure characteristic of Mtb populations necessitates carefully designed sampling strategies to capture the full spectrum of global genetic diversity. Robust pangenome analyses should incorporate representatives from all major phylogenetic lineages, as each lineage carries distinct genetic signatures and evolutionary adaptations that enrich the species’ overall diversity. Ensuring balanced lineage representation is essential to avoid sampling bias that could distort estimates of core and accessory genome content in favor of overrepresented groups. Geographic diversity is equally crucial, as regional strain populations often possess unique genetic traits shaped by local transmission patterns and selective pressures (Chekesa et al., 2024).

### 5.3 Gene prediction and annotation for bacterial pangenomics

Achieving consistent gene identification across diverse genome assemblies remains a core challenge in bacterial pangenomics. Variations in gene calling algorithms and parameter settings can significantly affect downstream homology detection and ultimately shape inferred pangenome architecture. Contemporary studies primarily rely on four widely used gene prediction tools-Prodigal, Glimmer, GeneMarkS-2, and the more recent Balrog, each employing distinct algorithmic strategies tailored to specific bacterial features (Dimonaco et al., 2022; Horsfield et al., 2023). Among these, Prodigal (PROkaryotic DYnamic programming Gene-finding ALgorithm) has become the most widely adopted choice in bacterial pangenomics due to its speed, accuracy, and robust performance across diverse GC content ranges (Hyatt et al., 2010). Unlike traditional Hidden Markov Model approaches, Prodigal utilizes log-likelihood scoring and dynamic programming algorithms to ensure rapid and precise gene prediction. Its widespread adoption is evidenced in its integration into major annotation pipelines such as Prokka, and numerous pangenome construction tools (Seemann, 2014). In contrast, Glimmer uses interpolated Markov models to distinguish coding regions, offering complementary predictions particularly useful in complex genomic contexts, albeit at a higher computational cost (Delcher et al., 2007). GeneMarkS-2, employing species-specific inhomogeneous Markov chains, is advantageous for organisms with well-characterized phylogenies but similarly demands more computational resources (Lomsadze et al., 2018). Crucially, no single predictor performs optimally across all bacterial species. Performance varies with GC content, genome organization, and phylogenetic background, and tool choice can significantly influence the results (Dimonaco et al., 2022). This variability has important implications for pangenome studies, as comprehensive benchmarking studies have revealed that tool selection can impact pangenome architecture estimates, necessitating a careful evaluation of prediction accuracy for the specific bacterial groups under investigation.

Beyond gene calling, annotation consistency encompasses standardized functional assignment and database consistency. Prokka exemplifies a widely adopted pipeline that combines Prodigal for gene prediction with a hierarchical annotation strategy. This strategy involves BLAST+ searches against curated databases (UniProtKB, RefSeq) followed by HMMER3 searches against protein family databases (Pfam, TIGRFAMs) (Seemann, 2014). This hierarchical strategy enables rapid annotation while maintaining functional accuracy across diverse bacterial genomes, creating a standardized framework essential for downstream pangenomic analyses.

### 5.4 Homology detection and clustering

Once consistent genes are established, the next major challenge is accurate homology detection. This task has driven the development of increasingly sophisticated clustering algorithms, designed to balance sensitivity, scalability, and biological accuracy. Standard BLAST searches typically employ *e*-value thresholds ≤1e-5 (0.00001) to infer significant homology, with coverage requirements often set between 50% and 80% of the shorter sequence to ensure biologically meaningful alignments (Tettelin et al., 2005). Early approaches relied heavily on BLAST-based all-vs.-all comparisons, which offered high sensitivity but became computationally limited with the exponential growth of genomic datasets (Altschul et al., 1990; Tonkin-Hill et al., 2020). To address this, faster clustering tools emerged and prompted a paradigm shift toward heuristic algorithms that traded some sensitivity for substantial gains in performance. The introduction of rapid clustering algorithms marked a key breakthrough in scalability. CD-HIT, for example, pioneered the use of word-based filtering strategies and greedy incremental clustering, processing sequences by decreasing length and grouping them via efficient word counting rather than expensive pairwise alignments (Fu et al., 2012). Similarly, USEARCH introduced further algorithmic optimizations and indexing strategies that enhanced computational efficiency while maintaining reasonable clustering accuracy (Edgar, 2010). These approaches successfully enabled the analysis of datasets comprising millions of sequences, though their speed advantages came at the cost of reduced sensitivity for detecting distant homologs. Recognizing the limitations of simple sequence similarity in distinguishing biologically meaningful orthology from recent gene duplication events led to the development of more advanced graph-based approaches. OrthoMCL exemplifies this transition, combining conventional similarity searches with Markov clustering to resolve complex homology relationships, particularly valuable for organisms with intricate evolutionary histories (Li et al., 2003). While more biologically accurate, this method requires substantially greater computational resources. The contemporary frontier in sequence clustering has been defined by algorithms that achieve a combination of BLAST-level sensitivity with heuristic-level speed. MMseqs2 represents this new generation, employing indexing strategies and optimized alignment algorithms that enable linear-time clustering of massive protein datasets without compromising sensitivity (Steinegger and Söding, 2017). However, even advanced clustering algorithms face challenges when applied to Mtb genomes due to their highly repetitive architecture. PE/PPE multigene families present complex clustering decisions due to conserved N-terminal domains that can confound similarity-based detection, potentially leading to inappropriate merging of functionally distinct family members or artificial fragmentation of genuine orthologs. IS6110 insertion sequences introduce additional complexity through variable copy numbers between strains. These repetitive elements require careful optimization of clustering parameters: conservative thresholds may artificially inflate accessory genome estimates by fragmenting related sequences, while permissive thresholds risk masking genuine functional diversity within gene families (Espinoza et al., 2025). This example illustrates broader principles governing selection among algorithmic paradigms, which require careful consideration of the evolutionary characteristics underlying each bacterial system under study.

Highly conserved species like Mtb benefit from rapid clustering approaches that can efficiently process large datasets. At the same time, more divergent bacterial groups demand the enhanced sensitivity provided by orthology-aware or graph-based methodologies (Vernikos et al., 2015). Furthermore, the optimization of similarity thresholds and clustering parameters must reflect the specific evolutionary pressures shaping each bacterial lineage, as inappropriate parameter selection can either artificially fragment genuine gene families or inappropriately merge functionally distinct groups. The particular similarity criteria employed during homology detection fundamentally determine pangenome architecture estimates in Mtb. Clustering algorithms establish boundaries between orthologous and paralogous relationships through sequence identity thresholds, coverage requirements, and alignment parameters, directly influencing gene family partitioning. These methodological decisions create measurable consequences in MTBC pangenome studies: relaxed alignment thresholds (such as Panaroo’s default 70% identity for diverse gene families) can inappropriately group partially deleted genes with complete ones, while stringent criteria (≥90% identity with ≥75% coverage, as used for H37Rv validation) may fragment genuine orthologs, fundamentally altering estimates of core and accessory genome content (Behruznia et al., 2025; Marin et al., 2025). The mathematical relationship between clustering parameters and gene family boundaries thus creates a direct link between methodological choices and biological interpretations of pangenome structure.

### 5.5 Matrix generation and statistical characterization

The culmination of the pangenomic construction process requires the systematic transformation of homologous gene clusters into quantitative data structures suitable for statistical analysis. This step is more than a technical transformation; it constitutes the conceptual bridge between raw computational information and meaningful biological interpretations that underpin our understanding of bacterial evolution. The generation of presence-absence matrices constitutes the methodological core of this transformation. These matrices represent genomes as rows and gene families as columns, with binary indicators (1 or 0) denoting the presence or absence of each genetic element in each genome (Medini et al., 2005). However, this apparent binary simplicity masks considerable complexity arising from both biological and technical realities of genomic analysis. Fragmented genes, ambiguous clustering assignments, and variations in assembly quality introduce challenges that require sophisticated quality control frameworks to ensure the resulting matrices reflect genuine biological variation rather than technical artifacts (Tonkin-Hill et al., 2020). Contemporary pangenome pipelines have developed extensive pre- and post-processing scripts for quality control, including diagnostic plots for contamination detection and gene count validation to address these systematic sources of error (Tonkin-Hill et al., 2020).

The subsequent statistical characterization process reveals fundamental patterns of genomic organization through the classification of gene families according to their frequency distributions. The definition of the core genome, previously conceptualized as genes present in all analyzed strains, has evolved toward a spectrum of criteria recognizing the technical and biological realities of modern pangenomic analysis. The selection of presence thresholds for defining the core genome represents one of the most critical and controversial decisions in pangenome analysis. While the strict definition of 100% presence offers mathematical certainty, it frequently underestimates essential gene content due to technical limitations, including incomplete assemblies, annotation errors, or genuine but rare gene losses (Matthews et al., 2024). This rigidity has led to widespread adoption of more flexible thresholds, typically ranging from 95% to 99% presence, each with distinct implications for the resulting pangenome architecture. The 95% threshold, widely utilized in bacterial pangenome studies, maximizes capture of functionally essential genes while accommodating minor technical variations (Zhang et al., 2023). Studies have demonstrated that this threshold optimally represents essential gene content in various bacterial species, capturing critical genetic elements that might be erroneously excluded by stricter criteria (Segerman, 2012; Zhang et al., 2023). Conversely, 99% thresholds offer a compromise between inclusivity and conservation, proving particularly appropriate for species with high-quality sequencing and assembly (Page et al., 2015). The transition between these thresholds can result in reclassification of hundreds of genes, dramatically altering core genome size estimates and, consequently, inferences regarding species genomic plasticity, with some studies showing core genome estimates varying by factors of three or more depending on threshold selection (Matthews et al., 2024). This threshold sensitivity extends beyond the core genome toward characterization of the accessory genome. The introduction of “soft-core” (genes present in 95%–99% of strains) and “shell” (genes with intermediate frequencies) concepts provides more nuanced resolution of pangenome architecture, recognizing that gene frequency distributions form a continuum rather than discrete categories (Page et al., 2015). This graduated perspective reveals patterns of gene conservation reflecting differential selective pressures, complex evolutionary histories, and lineage-specific adaptations across multiple resolution levels of pangenome analysis (Hyun et al., 2022). The implications of these methodological decisions transcend mere technical categorization. In species such as Mtb, where genetic diversity is inherently limited, threshold selection can determine whether the pangenome appears highly conserved or moderately variable. A 100% threshold might suggest a core genome of merely 1,166 genes (Zakham et al., 2021), while a 95% threshold could expand this estimate to over 3,700 genes (Espinoza et al., 2025), representing a greater than three-fold difference that fundamentally alters our understanding of the pathogen’s essential biology. Similar patterns have been observed across multiple bacterial species where threshold choice dramatically impacts core genome size estimates (Park et al., 2019). Beyond categorical classification, modern statistical characterization of pangenomes incorporates sophisticated quantitative analyses revealing emergent properties of bacterial evolution. Power-law regression models facilitate the prediction of potential pangenome expansion, while rarefaction analyses assess the sufficiency of genomic sampling (Parmigiani et al., 2024). The integration of these analytical elements into a coherent framework requires not only technical rigor but also a deep understanding of the underlying biology. Presence-absence matrices, far from being mere computational abstractions, encode complex evolutionary histories where each binary entry represents millions of years of selective pressure, genetic drift, and adaptation (Maistrenko et al., 2020). Their careful statistical analysis, informed by appropriate methodological decisions, constitutes the foundation upon which we build our understanding of bacterial diversity and evolutionary potential.

### 5.6 Functional analysis and biological interpretation of *Mycobacterium tuberculosis* pangenome

Beyond the technical construction of the pangenome, its actual value lies in translating genomic data into biological meaning. Functional interpretation bridges the gap between raw genetic information and the broader questions that drive TB research: How does genetic variation influence drug resistance? What genes define virulence or host adaptation? Which components are essential for pathogen survival? By integrating gene frequency patterns with functional annotation, pangenome studies move from describing genetic architecture to uncovering the mechanisms that shape Mtb evolution and clinical behavior. This level of analysis enables the identification of conserved core functions, high-priority therapeutic targets, and lineage-specific traits relevant to transmission and disease progression. The following section will explore how the biological interpretation of the Mtb pangenome has led to transformative applications in molecular epidemiology, resistance prediction, and virulence research–demonstrating how this comprehensive genomic framework is redefining our strategies for understanding and controlling TB.

## 6 Applications and objectives of *M. tuberculosis* pangenome studies

The comprehensive characterization of genetic diversity through pangenome approaches has proven particularly effective for Mtb despite the species’ unique evolutionary constraints. In the absence of horizontal gene transfer, pangenome analyses capture genetic diversity by detecting gene presence/absence patterns resulting from characteristic evolutionary mechanisms. Single nucleotide polymorphisms creating frameshifts or premature stop codons, complete gene deletions, and large sequence polymorphisms translate into detectable coding sequence variations that clustering algorithms classify as absent or lineage-specific genes, effectively capturing the predominantly deletion-driven diversification characteristic of clonal bacterial species (Silva-Pereira et al., 2024; Behruznia et al., 2025).

**FIGURE 2:**
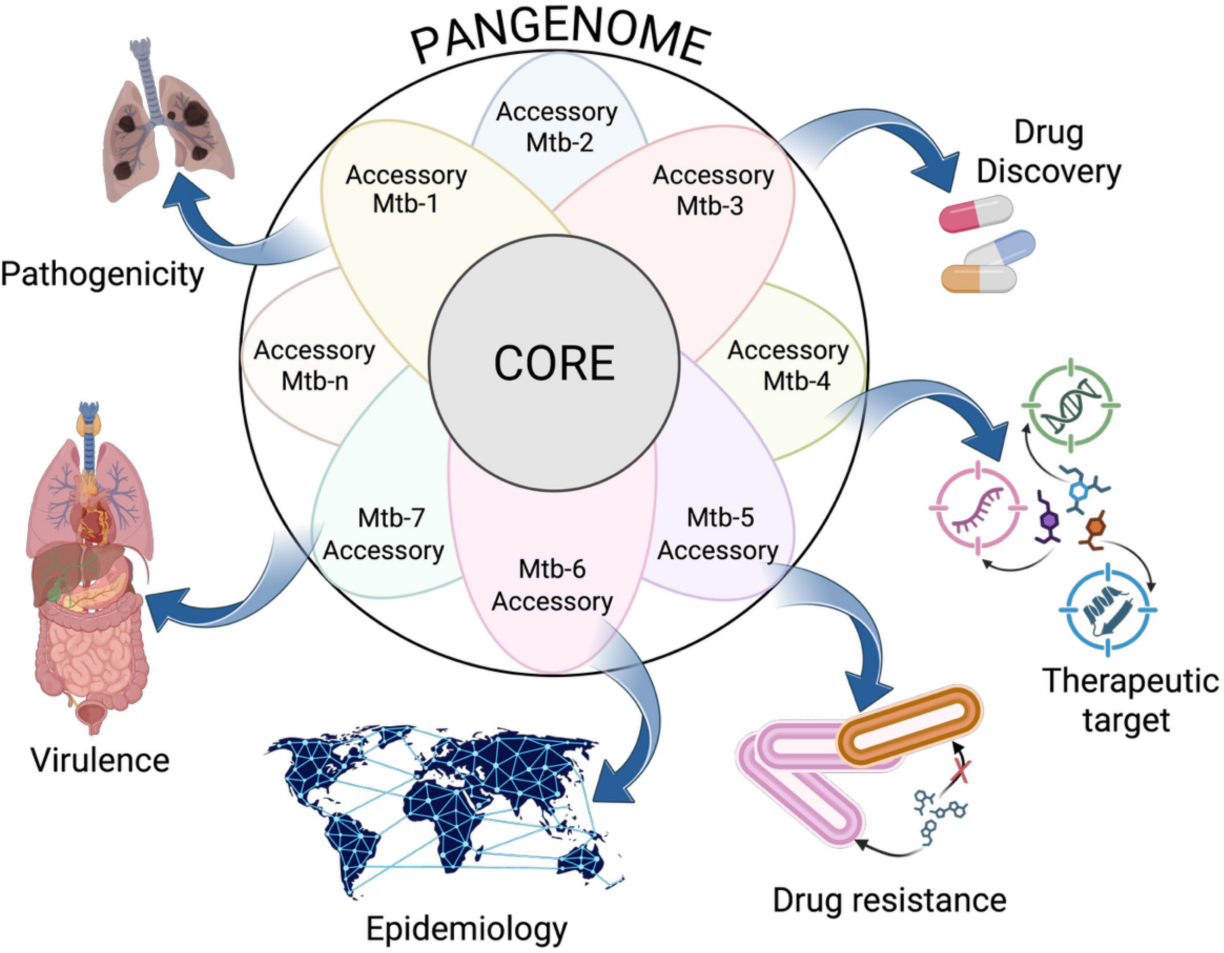
Pangenome applications in *Mycobacterium tuberculosis* studies. Pangenome analysis facilitates the assessment of pathogenicity and virulence by identifying a strain’s capacity to cause pulmonary infections or to infect other organs. It serves as a valuable tool for discovering antibiotic resistance and therapeutic targets within the *M. tuberculosis* pangenome, which encompasses DNA, RNA, and proteins. Epidemiological analysis utilizing pangenome aids in tracing transmission routes and understanding relationships between strains of varying origins. As the analysis progresses with the inclusion of more strain genomes (Mtb-1, Mtb-2, …, Mtb-n), the accessory genome may expand, uncovering strain-specific adaptations. Created in BioRender. Vázquez-Marrufo (2025) https://BioRender.com/kzarxxt.

This capacity to translate structural genomic changes into analyzable patterns of gene content has yielded transformative insights that are reshaping our understanding of TB biology, epidemiology, and clinical management (Figure 2). While early genomic studies focused on individual reference strains such as H37Rv, pangenome studies capture the full spectrum of genetic variation present across the global Mtb population, revealing previously hidden layers of complexity essential for understanding the pathogen’s success as a global health threat (Zhou et al., 2025). These applications extend across multiple domains of TB research and public health practice, from enhancing our ability to identify novel therapeutic targets to addressing the evolving challenges posed by MDR and XDR TB strains (World Health Organization [WHO], 2024; Dheda et al., 2024).

The following sections examine the key applications and objectives of Mtb pangenome studies, demonstrating how this approach is contributing to advances across the spectrum of TB research.

### 6.1 Molecular epidemiology

Pangenome analysis has enhanced molecular epidemiological investigations of TB by providing a comprehensive genomic context for understanding lineage-specific characteristics and regional strain diversity. The comprehensive genetic repertoire captured through pangenome studies enables researchers to identify lineage-specific markers and transmission patterns, complementing existing molecular typing approaches (Zhou et al., 2025). While traditional molecular typing methods, such as spoligotyping and MIRU-VNTR, provide robust discrimination for strain differentiation, pangenome approaches offer supplementary insights, particularly valuable for understanding the genetic basis of broader epidemiological patterns, such as lineage-specific geographic distributions, regional genetic adaptations, and population-level genetic signatures. Building on this foundation, specialized computational methods have been developed. The introduction of methods like PANPASCO exemplifies this advancement, using pangenome-based read mapping against reference genomes from major lineages to classify strains and identify population-level genetic patterns, facilitating lineage-specific epidemiological analysis (Jandrasits et al., 2019). Also, pangenomic approaches have uncovered critical genomic features, such as deletions in accessory genes associated with increased virulence in specific lineages, particularly the modern Beijing sub-lineage, which may contribute to higher transmissibility and drug resistance (Rufai et al., 2020). Moreover, by analyzing accessory genome components, it is possible to identify unique genetic signatures associated with specific geographical regions, population groups, or detect lineage-specific genes that correlate with particular epidemiological patterns or host adaptations (Chekesa et al., 2024). Pangenome association studies have extended this concept by directly correlating genetic variation with disease prevalence, exemplified by the identification of lineage-4 genes associated with TB patterns in specific Colombian populations (Hurtado-Páez et al., 2023). Collectively, these regional pangenomic approaches have consistently identified geographical patterns in accessory genome content and lineage-specific genetic signatures, demonstrating the value of population genetic characterization for understanding regional Mtb diversity (Morey-León et al., 2025).

### 6.2 Drug resistance characterization and therapeutic target discovery

Pangenome analysis has revolutionized the understanding of antimicrobial resistance in Mtb by providing comprehensive insights into both resistance mechanisms and novel therapeutic opportunities. This approach extends far beyond the analysis of well-characterized core resistance genes to encompass the entire genetic repertoire that may contribute to drug resistance phenotypes and therapeutic vulnerability. By analyzing the collective genetic diversity of resistant strains, it has been possible to identify novel resistance mechanisms, compensatory mutations, and epistatic interactions that influence treatment outcomes and resistance evolution (Kavvas et al., 2018). The integration of machine learning and structural analysis with pangenome data has significantly advanced the identification of genetic signatures of antimicrobial resistance and the prediction of resistance phenotypes from genomic sequences. These computational approaches have revealed complex epistatic interactions that contribute to resistance development and provided mechanistic insights into how resistance mutations affect protein function and bacterial fitness. Specifically, Kavvas et al. (2018) developed a computational platform that combines machine learning with genetic interaction analysis and 3D structural mutation mapping to identify antimicrobial resistance signatures in Mtb, revealing 97 epistatic interactions across 10 resistance classes and providing detailed structural insights into resistance mechanisms.

Advancing these machine learning approaches further, recent developments have addressed a fundamental limitation in molecular drug susceptibility testing: the reliance on single reference genomes that may miss resistance variants. Bahk et al. (2024) developed a pan-lineage reference genome (“MtbRf”) by systematically assembling previously unmapped reads from 3,614 Mtb genomes across major lineages, recovering genetic content absent from the standard H37Rv reference. This comprehensive reference genome improved drug susceptibility prediction accuracy by capturing resistance-associated variants that were previously undetectable using traditional single-strain references. The integration of these additional genetic sequences with machine learning algorithms demonstrated enhanced predictive performance across eight major anti-tuberculosis drugs, highlighting how pangenomic approaches can overcome the inherent bias of reference genome-based resistance detection methods.

Leveraging the comprehensive genetic landscape provided by pangenome studies, researchers have systematically identified essential genes within the core genome that represent high-priority therapeutic targets. The analysis of gene essentiality across diverse Mtb strains provides valuable insights into which genetic elements are indispensable for survival and pathogenesis, making them attractive candidates for drug development (Periwal et al., 2015; Dar et al., 2020). Recent advances have demonstrated the power of integrating pangenome analysis with subtractive proteomics and computational drug design, successfully identifying promising therapeutic targets such as isocitrate lyase and pantothenate synthetase, along with potential lead compounds including dihydroergotamine and abiraterone acetate (Khan et al., 2024). This systematic approach ensures that potential drug targets are assessed in the context of the complete genetic diversity observed among clinical isolates. The clinical translation of pangenomic insights has been further enhanced by advances in long-read sequencing technologies, which also improve diagnostic accuracy for drug-resistant strain identification by capturing structural variants and repetitive elements that remain undetectable through short-read approaches (Carandang et al., 2025). The combination of pangenome-informed variant databases with these sequencing platforms enables clinically actionable results, particularly in multidrug-resistant contexts where precise genetic characterization is critical for treatment decisions.

Beyond core genome targets, the accessory genome components revealed through pangenome analysis harbor genes encoding strain-specific virulence factors or metabolic pathways that could serve as targeted therapeutic opportunities. This expanded target space enables the development of personalized treatment strategies based on the genetic profile of individual clinical isolates, potentially improving treatment efficacy and reducing the likelihood of resistance development (Espinoza et al., 2025). Such personalized approaches represent a significant advancement over traditional one-size-fits-all treatment regimens.

### 6.3 Virulence and pathogenicity studies

The application of pangenomic approaches to virulence research has revealed critical insights into the genetic determinants underlying strain-specific differences in Mtb pathogenicity that remain undetectable to single-genome studies, addressing fundamental questions about why certain strains cause pulmonary (PTB) versus extrapulmonary disease (EPTB), exhibit enhanced transmissibility, or display distinct host adaptation patterns across diverse clinical contexts. Negrete-Paz et al. (2023) reported the use of pangenome reconstruction as a tool to reveal genomic features associated with strain clinical phenotypes. The analysis reported distinct genetic signatures associated with different clinical manifestations of the disease, with many of these signatures involving members of specialized gene families that have emerged as key players in TB pathogenesis (Negrete-Paz et al., 2023). Among the most prominent of these genetic determinants are the PE/PPE multigene families, comprising approximately 10% of the Mtb genome with 176 open reading frames (Akhter et al., 2012). These proteins, characterized by conserved proline-glutamate (PE) or proline-glutamate (PPE) motifs at their N-terminus, represent one of the most intriguing aspects of the Mtb genome, with various lines of evidence implicating selected family members in mycobacterial virulence (Fishbein et al., 2015). Pangenomic analyses have revealed that 81 core PE/PPE, virulence factor, and antigen genes are related to the thick, lipid-rich cell envelope phenotype of Mtb, including seven genes involved in maintaining cell wall integrity and cell morphotype, 16 genes for host-cell entry, and 32 genes associated with Mtb hypervirulence (Yang et al., 2018). Additionally, 112 core PE/PPE, virulence factor, and antigen genes are related to intracellular survival phenotype, encompassing 21 genes involved in stress response,18 genes affecting the antimicrobial activity of the phagosome, and 16 genes involved in nutrient absorption (Yang et al., 2018). This systematic characterization reveals the genetic basis underlying strain-specific pathogenic potential, providing insights that may help explain clinical diversity and prompting more sophisticated evolutionary analyses to understand the selective pressures shaping Mtb pathogenicity.

Building upon these pangenomic insights, Bundhoo et al. (2024) conducted a comprehensive molecular evolutionary analysis of core genes among 264 Mtb strains, determining the estimated rates of molecular evolution of select biological processes and molecular functions using the dN/dS ratio–a measure that compares the rate of amino acid-changing mutations to silent mutations, indicating evolutionary pressure on genes (Bundhoo et al., 2024). This evolutionary approach has been complemented by advanced pangenomic methodologies that challenge traditional notions of gene essentiality. A recent study demonstrated that 74% of core genes were deemed non-essential *in vitro*, with 38% supporting pathogen survival *in vivo*, suggesting the need to broaden current perspectives on gene essentiality and highlighting how strain-specific genetic profiles may influence treatment responses and clinical outcomes in diverse patient populations (Espinoza et al., 2025). While these findings have reshaped our understanding of core genome functionality, complementary analyses of the accessory genome components have provided critical insights into lineage-specific virulence mechanisms and adaptive strategies. Pangenomic analysis of modern Beijing sublineages revealed specific deletions in accessory genome sequences, including the complete deletion of CRISPR-associated endoribonuclease cas1 (Rv2817c), cas2 (Rv2816c), and CRISPR type III-a/m tube-associated proteins, suggesting that specific lineages have evolved distinct genetic architectures that may contribute to their enhanced transmissibility and drug resistance characteristics.

### 6.4 Future perspectives and challenges

While pangenome studies have yielded significant insights into Mtb genetic diversity and resistance mechanisms, translating these findings into practical applications requires addressing key methodological and implementation challenges. The standardization of analytical methodologies represents a fundamental priority, requiring coordinated efforts that acknowledge both technical and practical realities. A pragmatic approach involves developing standardized benchmark datasets with well-characterized clinical isolates representing major lineages and resistance profiles, enabling systematic comparison of existing tools rather than enforcing single methodological approaches (Marin et al., 2025; Behruznia et al., 2025). Such initiatives could enhance reproducibility and comparability across research groups while addressing the methodological controversies highlighted throughout this review. The integration of pangenomic approaches into tuberculosis surveillance systems presents implementation challenges that vary significantly across different resource settings. While pangenomic analysis could enhance molecular epidemiological investigations and resistance monitoring in high-resource contexts, implementation in high-burden settings faces substantial barriers, including limited computational infrastructure, a shortage of trained personnel, and competing priorities for basic diagnostic capacity. Regional reference laboratories with pangenomic capabilities serving multiple countries, coupled with capacity-building partnerships, may represent a more feasible implementation strategy that acknowledges these resource disparities. The integration of pangenome data with complementary omics approaches offers promising avenues for understanding the functional significance of genetic diversity, yet presents challenges in data harmonization, computational scalability, and biological interpretation. Recent advances in machine learning applied to pangenomic data have produced increasingly accurate models for predicting drug resistance, with some approaches approaching the accuracy of traditional phenotypic testing while providing results in shorter timeframes (Kavvas et al., 2018; Bahk et al., 2024). However, demonstrating that such multi-layered approaches provide clinically actionable insights that justify their additional complexity compared to existing molecular diagnostic tools remains an ongoing challenge.

Several developments suggest a positive trajectory for the field. Emerging computational tools designed specifically for clonal pathogens like Mtb are addressing methodological limitations, while international collaborative initiatives continue to establish data sharing standards that facilitate cooperation. The expansion of global genome databases through initiatives like the CRyPTIC consortium provides increasingly comprehensive representations of Mtb genetic diversity. Cloud-computing platforms are beginning to democratize access to sophisticated analytical capabilities, potentially addressing infrastructure limitations in resource-constrained settings. Nevertheless, the clinical translation of pangenomic approaches requires rigorous validation through prospective studies demonstrating improved treatment outcomes. While preliminary evidence suggests potential for personalized treatment strategies based on genetic diversity information, further validation studies are needed to demonstrate the clinical utility and cost-effectiveness of pangenome-informed diagnostic and therapeutic approaches. Success in realizing the transformative potential of pangenomics for tuberculosis control will ultimately depend on sustained collaboration between research institutions, public health organizations, and clinical practitioners developing practical, validated tools that address clinical needs. The convergence of methodological advances, expanding databases, and international collaborative frameworks positions pangenomics for substantial contributions to global tuberculosis control efforts.

## 7 Conclusion

*Mycobacterium tuberculosis* pangenome represents a transformative framework for elucidating the genetic diversity underlying one of humanity’s most persistent pathogens. This review has explored the structural organization of the Mtb pangenome, the ongoing methodological debates regarding its classification as open or closed, and the systematic approaches required for robust pangenome construction. The expansion of pangenomic applications across molecular epidemiology, drug resistance characterization, and virulence studies underscores a paradigmatic shift from reference-centric to diversity-inclusive approaches in TB research. Although substantial challenges remain-particularly in standardizing analytical methodologies and integrating multi-omics datasets- the pangenomic perspective offers unprecedented insights into strain-specific adaptations, resistance mechanisms, and therapeutic targets that remain undetectable through single-genome analyses. Looking ahead, the convergence of pangenomic data with advanced machine learning approaches and functional validation strategies holds the potential studies will likely unlock new opportunities for TB treatment, enhanced surveillance systems, and novel therapeutic interventions in the ongoing battle against this global health threat.
